# Effectiveness of Cognitive Behavioral Therapy Provided Through a Web Application for Subthreshold Depression, Subthreshold Insomnia, and Subthreshold Panic: Open-Labeled 6-Arm Randomized Clinical Trial Pilot Study

**DOI:** 10.2196/63139

**Published:** 2025-02-03

**Authors:** Kayoko Taguchi, Mirai Miyoshi, Yoichi Seki, Shiori Baba, Eiji Shimizu

**Affiliations:** 1 Research Center for Child Mental Development Chiba University Chiba Japan; 2 Graduate School of Medicine Chiba University Chiba Japan; 3 Chiba University Hospital Chiba Japan

**Keywords:** minimally important change, nonguided cognitive behavioral therapy, subthreshold depression, subthreshold insomnia, subthreshold panic, cognitive behavioral therapy, CBT, psychiatric disease, primary care, interventions, depression, anxiety, insomnia, psychological therapy

## Abstract

**Background:**

A common definition of “subthreshold” is that the diagnostic threshold is not met but the individuals are not asymptomatic. Some symptoms are present, causing significant difficulty in functioning and negatively impacting quality of life. Despite the attention given to subthreshold symptoms and the interventions for subthreshold symptoms being efficient in preventing the transition to psychiatric disease in primary care, reports on specific interventions are insufficient.

**Objective:**

This study aimed to verify the effectiveness of internet-delivered cognitive behavioral therapy (ICBT) for subthreshold depression (SD), subthreshold insomnia (SI), and subthreshold panic (SP). Additionally, this study aimed to explore the minimally important change (MIC) of each subthreshold group’s effectiveness outcome.

**Methods:**

Participants aged 18-70 years from internet research monitors were categorized into SD, SI, and SP groups based on screening assessment. They were randomly assigned to intervention or control groups within each subthreshold symptom. The intervention groups worked on 4 weeks of nonguided ICBT (“Mentre”), while the control groups worked on a sham app. The primary outcome was the score change from screening (T1) to 4-week follow-up (T4) using the Center for Epidemiologic Studies Depression Scale (CESD) in the SD group, the Pittsburgh Sleep Quality Index (PSQI) in the SI group, and the Panic and Agoraphobia Scale (PAS) in the SP group. Secondary outcomes were score changes in the Generalized Anxiety Disorder-7 (GAD-7) scale, the Patient Health Questionnaire 9 (PHQ-9), the CESD, the PSQI, and the PAS, except the primary outcome in each group. Secondary outcomes were analyzed using complete-case analysis and repeated-measures ANOVA. Additionally, the MIC in the primary endpoint for each group was also calculated as an exploratory outcome.

**Results:**

The SD, SP, and SI groups contained 846, 597, and 1106 participants, respectively. In the SD group, the difference in the CESD score change from baseline to follow-up between the intervention and control groups was significant (difference=0.52, 95% CI 1.29-4.66, *P*<.001). In the SI group, the difference in the PSQI score change was also significant (difference=0.53, 95% CI 0.11-0.94, *P*=.01). However, in the SP group, the difference in the PAS score change was not significant (difference=0.07, 95% CI –2.00 to 2.15, *P*=.94).

**Conclusions:**

Our ICBT program Mentre contributes to the improvement of SI and SD. This suggests that nonguided ICBT may be effective in preventing SI and SD from progressing to the full threshold. However, appropriate definitions of subthreshold symptoms are necessary. In particular, it is difficult to define SP, and further research that considers the specific factors of each subthreshold symptom is necessary to accumulate evidence.

**Trial Registration:**

University Hospital Medical Information Network (UMIN) UMIN000051280; https://tinyurl.com/2wyahhe3

## Introduction

A common definition of “subthreshold” is that the diagnostic is not met but the individuals are not asymptomatic, that is, some symptoms are present, causing significant difficulty in functioning and negatively impacting the quality of life [1,2]. However, the concept of “subthreshold” symptoms remains unclear in the clinical setting. For instance, according to the latest scoping review of subthreshold depression (SD), there are many definitions based on the assessment tool cutoff point of the Center for Epidemiologic Studies Depression Scale (CESD), Beck’s Depression Inventory II (BDI-II), and the Patient Health Questionnaire 9 (PHQ-9) and on the duration of symptoms. As a result, these definitions “seem to be arbitrary, with considerable overlap” [3]. Even the most popular subthreshold symptom is vague in its definition, and the other subthreshold symptoms, which are little mentioned, are even more so.

Although the definition is ambiguous, intervening for subthreshold symptoms is efficient in preventing the transition to psychiatric disease in primary care. Research has already suggested that shortening the duration of untreated illness (DUI) has a good therapeutic effect. The DUI is “measured as the interval between onset of the disorder and when the patient receives the first adequate treatment for that psychiatric disorder” [4], and many studies have claimed that the DUI affects treatment prognosis [5]. Furthermore, some studies have revealed the risk of transition from the subthreshold level to disease [6]. There is consensus that early intervention can be applied flexibly in different formats and across different target groups [7,8]. For instance, since SD and subthreshold anxiety occur twice as often as depression and anxiety [9], so appropriate definitions are, of course, essential for a concrete approach to primary care [10]. Furthermore, insomnia symptoms are closely associated with SD and subthreshold anxiety, and prevention programs for subthreshold insomnia (SI) have been reported to not only decrease depressive symptoms but also improve insomnia [11]. Namely, when considering primary care for subthreshold symptoms, overlapping symptoms also need to be considered; as a result, the treatment for specific subthreshold symptoms should also have the potential to cover a wide range of subthreshold symptoms. However, although there are some reports regarding SD interventions, there are insufficient studies on specific interventions in the subthreshold stage.

With limited reports, cognitive behavioral therapy (CBT) has been shown to be effective for subthreshold symptoms. CBT, which is a type of psychological therapy, improves symptoms by understanding patterns of maladaptive thinking and behavior and correcting the vicious cycle that perpetuates symptoms. It has been shown to be effective in improving diagnosed depression, anxiety, and insomnia [12-14]. However, nonguided internet-delivered cognitive behavioral therapy (ICBT) also has been shown to be effective for SD in a latest meta-analysis [15]. Since subthreshold symptoms are not severe, it is preferable that interventions be relatively short, easily accessible, and cost-effective. Nonguided CBT is more scalable and affordable [16,17], and it is suitable for people who have subthreshold symptoms but have no motivation to visit a hospital. However, there is insufficient evidence for ICBT as a subthreshold symptom treatment, and only 1 or 2 randomized controlled trials (RCTs) have verified that ICBT has valid effects on SD, SI, and subthreshold panic (SP). SI treatment verification over the internet has rarely been reported [18,19]. There are few reports on subthreshold anxiety, particularly reports on SP, which are mostly epidemiology and fact-finding surveys. Among the few papers on this topic, agreement has been reached concerning early intervention being valid and cost-effective for SP; however, they are not ICBTs [20,21].

Additionally, there are discussions of how patients perceive the significance of receiving ICBT for subthreshold symptoms. In recent years, interesting research results and suggestions concerning the minimally important change (MIC) have been reported [22]. The MIC is the smallest change or difference that patients perceive as significant. Researchers who reported on the MIC noted that the only satisfactory approach is for patients themselves to measure the health conditions they experience and know best. It is essential to discuss whether the MIC has implications for the significance of early intervention for subthreshold symptoms.

This study aimed to verify the effectiveness of the “Mentre” program as an ICBT for SD, SI, and SP in a 6-arm RCT. Additionally, this study aimed to explore the MIC of each subthreshold group’s effectiveness outcome. These results will be useful as the first evidence accumulated about ICBT for subthreshold symptoms with stricter criteria and for knowledge of the MIC among patients with subthreshold symptoms.

## Methods

### Ethical Considerations

This study was conducted with the approval of the Institutional Review Board of Chiba University Hospital (approval number: G2022005). Additionally, the Clinical Research Ethics Review Committee oversaw proper implementation of the study at least once a year.

An explanatory document was presented to each web program site, and consent was obtained. Participants were informed that participation was voluntary, that they could decline at any time for any reason, and that there would be no negative consequences for declining. Additionally, participants could withdraw their consent after providing informed consent.

For privacy, all participants’ data were anonymized. Participants were granted points that would enable them to shop online as compensation for research participation. Each intervention group received 1000 points if the participants worked on the program and answered the outcome inventory completely, while the control groups received 800 points.

### Trial Design and Procedure

The study was a prospective 6-arm, parallel-group RCT (Multimedia Appendix 1) [23]. The 6 arms were classified as SD, SI, and SP groups with 2 arms each. The trial was registered as “Effect of Mental Training Web Application for Subthreshold Insomnia, Subthreshold Depression and Subthreshold Panic Six Arms Randomized Controlled Trial” with the University Hospital Medical Information Network (UMIN; registration number: UMIN000051280). Participants were recruited from the internet research monitor of the NTT Com Online Marketing Solutions Corporation in May 2023. Participants who consented to the study completed a screening evaluation (T1) and a symptom persistence evaluation (T2) after 2 weeks. They were classified as having SD, SI, or SP and allocated to intervention or control groups. The intervention was a 4-week ICBT program Mentre, while the control program was a sham app, both of which started in June 2024 for each group. All participants were sent reminder emails every weekend to increase motivation and encourage continuous practice. After the program was complete, the participants were assessed for their symptoms postevaluation (T3), and 4 weeks later, follow-up evaluations were carried out (T4). Participants were awarded points for participating in the study and answering each time (T1-T4).

### Participants

Participants were aged 18-70 years and did not visit a hospital for any disease. The exclusion and inclusion criteria for this study were as follows:

- Individuals who experienced little interest in things or had depressed thoughts almost every day for 2 weeks and met more than 5 items of depressive symptoms in the PHQ-9 were excluded as having depression. Of those who were not excluded, individuals with a CESD score of 16 or higher were eligible as those with SD.
- Individuals with SI who had PSQI scores greater than 6 and insomnia symptoms lasting less than 3 months were also eligible.
- Individuals with SP who had Panic and Agoraphobia Scale (PAS) scores greater than 9 and who experienced anxiety-related panic symptoms continuously lasting less than 1 month were also eligible. In the case of duplicate symptoms, if the panic score met the eligibility criteria, participants were allocated to the SP group preferentially because panic symptoms are more specific than depression and anxiety symptoms. SD sometimes overlaps with SI; hence, participants who showed SD and SI symptoms were allocated to the SD group.
- The exclusion criteria included individuals with a history of diagnosed depression, insomnia, or panic disorder and those who experienced difficulty or extreme difficulty in daily life owing to anxiety symptoms on the Generalized Anxiety Disorder-7 (GAD-7) scale or depressive thoughts on the PHQ-9.

### Interventions

The intervention program Mentre was based on ICBT. We prepared 3 types of Mentre programs for each subthreshold symptom: SD, SI, and SP. Participants in the intervention groups undertook the relevant program for 4 weeks. The basic structure involved participants undergoing the first session at the beginning of the week and completing their homework throughout the remainder of the week. The control groups similarly recorded daily activities and weather conditions (sham app) in all subthreshold groups. All participants received guidance via email and accessed their program website through the URL provided on their mobile phones, personal computers, and tablets. The SD program included (1) cognitive restructuring about irrational thoughts, (2) psychoeducation for a depressive mood, (3) rumination distraction, and (4) behavioral activation to positive behavior. The SI program included (1) a sleep diary, (2) a review of one’s sleeping behavior, (3) a review of one’s sleep cognition, and (4) a review of one’s sleep efficiency and sleep restriction methods. The SP program included (1) learning concerning panic disorder, (2) attention shift training for when facing a panic attack, (3) how to change the worst image of panic to a rational image, and (4) how to stop avoidant behavior. Each program involved paying attention to become aware of symptoms and to change irrational thoughts to rational thoughts and negative behavior to positive behavior. Participants could inquire about this study through each web program, and the researcher and manager of the web program responded accordingly. Reminder emails were sent to all participants at the end of each week to continue and not to forget working on the program.

### Outcomes

#### Primary Endpoints

We set separate primary endpoints for each subthreshold symptom for the SD, SI, and SP groups: score change from screening (T1) to 4-week follow-up (T4) in each group:

- CESD in the SD group: The CESD is a brief self-report questionnaire for measuring the severity of depressive symptoms. It consists of 20 questions assessing numerous symptoms of depression experienced in the past week, with most items focusing on the emotional component of depression. Scores range from 0 to 60 points, with higher scores indicating more severe depressive symptoms. It has been used in many clinical trials involving a wide range of age groups [24,25].
- The Pittsburgh Sleep Quality Index (PSQI) in the SI group: The PSQI is a self-assessment consisting of 19 questions across 7 subscales (sleep quality, sleep latency, sleep duration, habitual sleep efficiency, sleep disturbances, hypnotic medication use, and daytime dysfunction). The total score indicates sleep quality and ranges between 0 (good sleep quality) and 21 (very poor sleep quality) points. The PSQI has been validated as a reliable and valid measure of subjective sleep quality in clinical practice and experimental studies [26,27].
- PAS in the SP group: The PAS is a 13-item questionnaire assessing panic attacks, composed of 5 factors (fear behavior, avoidance behavior, anticipatory anxiety, degree of disability, and health concerns) and evaluates the condition over the past week. Each item is scored from 0 to 4 points, with higher scores indicating more severe symptoms [28].

#### Secondary Endpoints

The secondary endpoints were the CESD, PSQI, and PAS score changes from screening (T1) to postevaluation (T3) and 4-week follow-up (T4) in all groups, as well as subsequent questionnaires. Additionally, the MIC in the primary endpoint for each group was also calculated.

- GAD-7 scale: It has been shown to have reliability, criterion, construct, factorial, and procedural validity. Cutoff points optimizing sensitivity (89%) and specificity (82%) were identified. The scale has 7 items assessing the severity of GAD in the past 2 weeks on a 4-point Likert scale (0=not at all, 1=1 episode, 2=on half or more days, and 3=almost daily). The minimum and maximum scores are 0 and 21, respectively (0-4, 5-9, 10-14, and 15-21 indicate no, mild, moderate, and severe symptoms, respectively). The cutoff score for clinically significant symptoms of anxiety is 10 [29].
- PHQ-9:It has diagnostic validity (for the diagnosis of 1 or more PHQ disorders; κ=0.65; overall accuracy=85,; sensitivity=75%, specificity=90%). It consists of 9 items scored on a 4-point Likert scale (0=not at all, 1=on several days, 2=on half or more days, and 3=almost daily). The minimum and maximum scores are 0 and 27, respectively (0-4, 5-9, 10-14, 15-19, and 20-27 indicate no, mild, moderate, moderate-to-severe, and severe symptoms, respectively). The PHQ cutoff score for clinically significant depressive symptoms is 10 [30].

### Exploratory Endpoints

#### Minimally Important Change

The MIC is the minimum change threshold that the patient feels has improved or worsened and the change that is considered significant to the patient [20,29]. When patients evaluate their treatment effect using certain measures, it remains unclear how much change is meaningful. Hence, many studies suggest considering the MIC concept [32,33].

### Sample Size

The sample size was calculated as follows: We needed to consider each subthreshold symptom, but there was insufficient evidence to estimate the effect size. We estimated the effect size of nonguided ICBT to be 0.2 based on a previous study [34]. Assuming an α level of .05 and a β level of .20, analysis was performed, resulting in a calculated sample size of 82. Based on the meta-analysis, more than 50% of the participants were expected to drop out. Hence, the sample size was set at 160 for each of the intervention and control groups.

### Registration and Randomization

For case registration, participants who met the criteria were automatically registered after a preliminary screening on the server of an internet research company (NTT Com Online Marketing Solutions Corporation. This company assigned participants to the SI group, SD group, or SP group and further randomly assigned them to either the intervention or the control subgroup. The assigned control factors were CESD score≥23 or not for SD, PSQI score≥12 or not for SI, and PAS score≥22 or not for SP; sex was also assigned as a control factor for all groups. Participants were informed via email or other means about how to access the corresponding intervention program.

### Protocol Deviations

Participants were provided the Mentre program through the web; therefore, dropouts and loss to follow-up were expected as main deviations. The number of not-evaluable cases was recorded. If any case reported a deviation due to an emergency crisis, the researcher immediately reported this to the Clinical Research Ethics Review Committee.

### Statistical Methods

#### Main Analysis

Baseline variables were compared using the Fisher exact test and an unpaired 2-tailed *t* test for categorical and continuous variables, respectively. The significance level was set at .05. For the main analysis of treatment effects, the means of the least squares and their 95% CIs were estimated by repeated-measures ANOVA with each change in the CESD, PSQI, and PAS scores at the 4-week follow-up for each group. The model included intervention group, time, and intervention-by-time interaction as fixed effects. All comparisons were planned, and all *P* values were 2-sided. *P*<.05 was considered statistically significant. The primary outcome formed the basis of complete case analysis. No special complementary processing by statistical methods was performed for missing values. All statistical analyses were performed using R version 4.3.1 (R Foundation for Statistical Computing).

#### Secondary Analysis

To examine the MIC, we used an anchor. The patient-reported outcome (PRO) scores for each primary outcome were classified into the following anchor categories: “much better,” “slightly better,” “about the same,” “slightly worse,” and “much worse.” The average PRO score classified as “slightly better” was considered the MIC. We calculated the CESD, PSQI, and PAS scores of the MIC in this study.

## Results

### Participant Details

**Figure 1:**
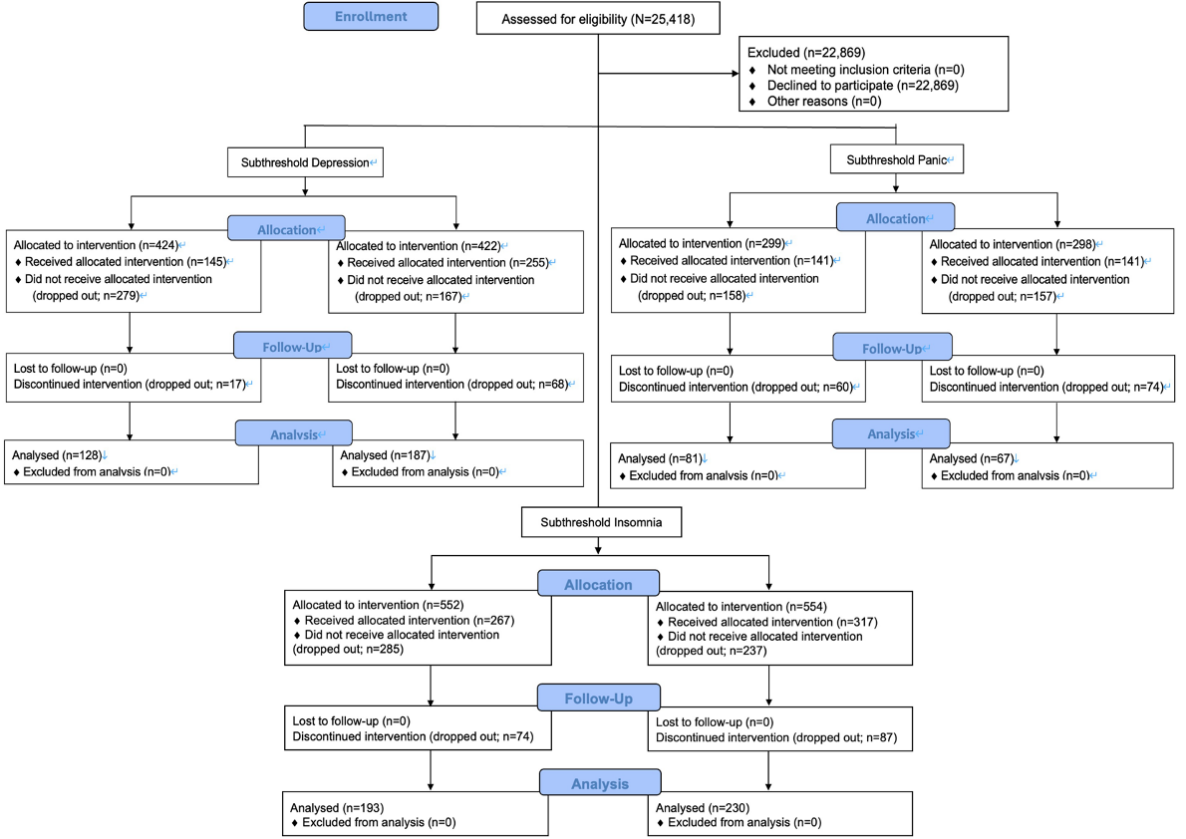
Flow diagram of the study participants.

The flow of participants through the study is illustrated in Figure 1. This study was announced to 215,000 internet research monitors, and 25,418 (11.8%) monitors provided consent. Of them, 2549 (10%) participants with SD, SI, or SP symptoms were screened (T1) and assigned to 1 of 6 arms. Afterward, 2091 (82%) participants’ symptoms continued after 2 weeks (T2). In total, 846 (33.2%) participants (n=424, 50.1%, intervention vs n=422, 49.9%, control participants) were classified as having SD, 1106 (43.4%) participants (n=552, 49.9%, intervention vs n=554, 50.1%, control participants) as having SI, and 597 (23.4%) participants (n=299, 50.1%, intervention vs n=298, 49.9%, control participants) as having SP according to screening evaluation scores. A total of 145 (34.2%) of 424 participants who were assigned to the SD group undertook the Mentre program, with 17 (11.7%) dropping out during the intervention. A total of 267 out of 552 participants who were assigned to the SI group undertook the Mentre program, with 74 dropping out during the intervention (27.7%). A total of 141 out of 299 participants who were assigned to the subthreshold panic group undertook the Mentre program, with 60 dropping out during the intervention (43.2%).

Table 1 presents the clinical characteristics of the patients in each group. In all 3 groups, no significant difference was found between the intervention and control subgroups in any average value, including sex, age, marital status, cohabitation rate, employment status, and income.

**Table 1:** Baseline demographic and clinical characteristics (N=2549).

Characteristics		SD^a^				SI^b^				SP^c^		
		Intervention (n=424)	Control (n=422)	*P* value	Intervention (n=552)		Control (n=554)	*P* value	Intervention (n=299)		Control (n=298)	*P* value
Sex (male), n (%)		130 (30.7)	130 (30.8)	.99	225 (40.7)		227 (41.0)	.951	106 (35.4)		107 (35.9)	.932
Age (years), mean (standard deviation)		41.3 (12.48)	42.5 (11.68)	.068	42.3 (11.74)		42.8 (12.10)	.546	41.2 (12.60)		42.1 (12.03)	.363
**Marital status, n (%)**												
	Married	229 (54.0)	239 (56.6)	.448	318 (57.6)		331 (59.7)	.502	154 (51.5)		156 (52.3)	.870
	Living with someone	322 (75.9)	317 (75.1)	.811	425 (77.0)		429 (77.4)	.886	236 (78.9)		229 (76.8)	.555
	Education, mean (standard deviation)	14.8 (1.99)	14.7 (2.05)	.410	14.9 (1.95)		14.7 (1.92)	.059	14.6 (2.10)		14.3 (2.15)	.208
	Employment, n (%)	284 (66.9)	291 (68.9)	.556	416 (75.3)		402 (72.6)	.304	195 (65.2)		186 (62.4)	.496
**Yearly income (yen), n (%)**		—^d^	—	.783	—		—	.320	—		—	.150
	<1,000,000 (US $6322.53)^e^	131 (30.8)	119 (28.2)	—	126 (22.8)		143 (25.8)	—	105 (35.1)		112 (37.6)	—
	1,000,001-5,000,000 (US $6322.54-$31,612.66)	203 (47.8)	214 (50.7)	—	284 (51.4)		268 (48.3)	—	137 (45.8)		135 (45.3)	—
	5,000,001-10,000,000 (US $31,612.66-$63,225.31)	78 (18.4)	75 (17.7)	—	128 (23.2)		121 (21.8)	—	53 (17.7)		40 (13.4)	—
	>10,000,000 (US $63,225.31)	12 (2.8)	14 (3.3)	—	14 (2.5)		22 (4)	—	4 (1.3)		11 (3.7)	—

^a^SD: subthreshold depression.

^b^SI: subthreshold insomnia.

^c^SP: subthreshold panic.

^d^Not applicable.

^e^An exchange rate of 1 yen=US $0.0063 has been applied.

### Primary Outcome

**Figure 2:**
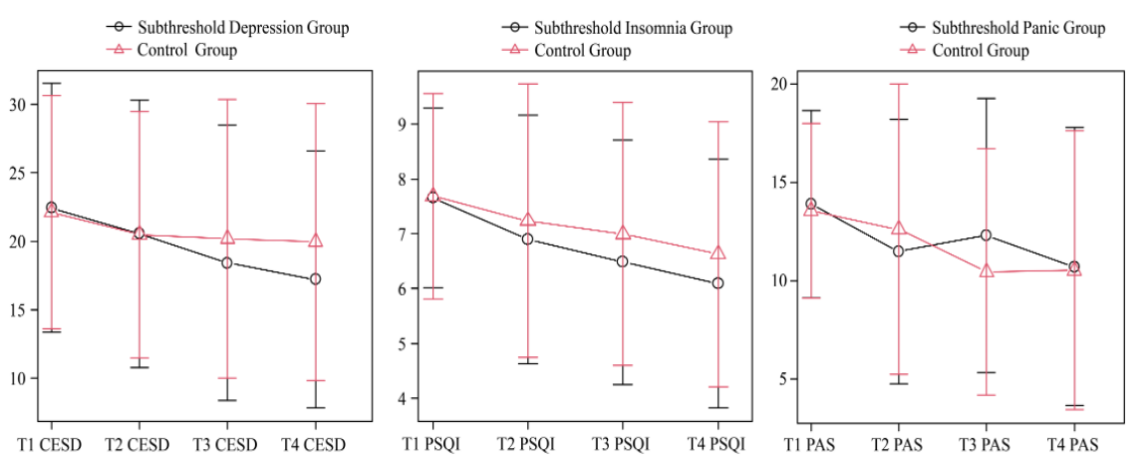
The effect of the Mentre program as the primary outcome for each group.

Tables 2-4 and Figure 2 show the effects of the Mentre program as the primary outcome for each group. In the SD group, differences in the CESD score change from T1 to T4 between the intervention and control groups were significant (difference=0.52, 95% CI 1.29-4.66, *P*<.001). In the SI group, differences in the PSQI score change from T1 to T4 between the intervention and control groups were significant (difference=0.53, 95% CI 0.11-0.94, *P*=.01). In the SP group, differences in the PAS score change from T1 to T4 between the intervention and control groups were not significant (difference=0.07, 95% CI –2.00 to 2.15, *P*=.94).

**Table 2:** Primary outcome ANOVA results for the SD^a^ group using the CESD^b^.

Week^c^	Intervention (n=128), mean (standard deviation)	Control (n=187), mean (standard deviation)	Estimate (95% CI)	SE	*P* value	Cohen's d
T1	22.42 (9.03)	22.11 (8.48)	—^d^	—	—	—
T2	20.54 (9.70)	20.47 (8.97)	0.16 (–1.33 to 1.65)	0.76	.829	—
T3	18.41 (9.98)	20.18 (10.14)	2.01 (0.28 to 3.73)	0.88	.023^e^	0.18
T4	17.20 (9.33)	19.94 (10.05)	2.98 (1.29 to 4.66)	0.86	.001^f^	0.28

^a^SD: subthreshold depression.

^b^CESD: Center for Epidemiologic Studies Depression Scale.

^c^T1: screening; T2: symptom persistence evaluation; T3: postevaluation; T4: 4-week follow-up.

^d^Not applicable.

^e^*P*<.05.

^f^*P*<.01.

**Table 3:** Primary outcome ANOVA results for the SI^a^ group using the PSQI^b^.

Week^c^	Intervention (n=193), mean (standard deviation)	Control (n=230), mean (standard deviation)	Estimate (95% CI)	SE	*P* value	Cohen's d
T1	7.66 (1.63)	7.69 (1.87)	—^d^	—	—	—
T2	6.90 (2.26)	7.23 (2.49)	0.32 (–0.06 to 0.69)	0.19	.099	—
T3	6.48 (2.23)	7.00 (2.40)	0.50 (0.10 to 0.90)	0.20	.015^e^	0.22
T4	6.09 (2.26)	6.63 (2.42)	0.53 (0.11 to 0.94)	0.21	.013^e^	0.23

^a^SI: subthreshold insomnia.

^b^PSQI: Pittsburgh Sleep Quality Index.

^c^T1: screening; T2: symptom persistence evaluation; T3: postevaluation; T4: 4-week follow-up.

^d^Not applicable.

^e^*P*<.05.

**Table 4:** Primary outcome ANOVA results for the SP^a^ group using the PAS^b^.

Week^c^	Intervention (n=81), mean (standard deviation)	Control (n=67), mean (standard deviation)	Estimate (95% CI)	SE	*P* value	Cohen's d
T1	13.89 (4.73)	13.54 (4.40)	—^d^	—	—	—
T2	11.47 (6.68)	12.60 (7.32)	1.41 (–0.54 to 3.36)	0.99	.156	—
T3	12.30 (6.93)	10.43 (6.21)	–1.65 (–3.62 to 0.33)	1.00	.101	0.29
T4	10.69 (7.02)	10.52 (7.05)	0.07 (–2.00 to 2.15)	1.05	.945	0.02

^a^SP: subthreshold panic.

^b^PAS: Panic and Agoraphobia Scale.

^c^T1: screening; T2: symptom persistence evaluation; T3: postevaluation; T4: 4-week follow-up.

^d^Not applicable.

### Secondary Outcomes

Tables 5-7 present the means of the secondary endpoints in each group and the differences between groups. In the SD group, differences in the mean PSQI and PAS scores at either time point were not significant; however, the difference in the PHQ-9 score at T4 was significant (difference=1.00, 95% CI 0.04-1.97, *P*=.04). Furthermore, at the time of postevaluation (T3), a significant difference was revealed in the PAS score (difference=2.05, 95% CI 0.02-4.08, *P*=.047). In the SI group, at the time of postevaluation (T3), a significant difference was found in the PSQI (difference=0.50, 95% CI 0.01-0.90, *P*=.02); however, between-group differences in changes in other endpoints were not significant at any time point. In the SP group, between-group differences in changes in all secondary endpoints were not significant.

**Table 5:** Secondary outcome ANOVA results for the SD^a^ group.

Variables and weeks^b^			Intervention, mean (standard deviation)	Control group, mean (standard deviation)		SE (95% CI)		*P* value	Cohen's d
**PSQI** ^c^ **(intervention: n=128; control: n=187)**									
	T1	6.01 (2.13)		6.47 (2.44)	—^d^		—		—
	T3	5.83 (2.57)		6.53 (2.82)	0.26 (–0.12 to 0.89)		.13		0.26
	T4	5.52 (2.48)		5.97 (3.13)	0.28 (–0.41 to 0.67)		.64		0.16
**PAS** ^e^ **(intervention: n=38; control: n=48)**									
	T1	4.29 (3.70)		3.79 (2.21)	—		—		—
	T3	4.24 (4.56)		5.94 (5.39)	1.02 (0.02 to 4.08)		.05		0.34
	T4	4.29 (5.34)		4.60 (5.36)	1.09 (–1.50 to 2.84)		.54		0.06
**PHQ-9** ^f^ **(intervention: n=128; control: n=187)**									
	T1	7.81 (4.42)		7.80 (4.19)	—		—		—
	T3	6.57 (5.18)		6.83 (5.23)	0.45 (–0.61 to 1.17)		.54		0.05
	T4	5.69 (5.53)		6.68 (5.30)	0.49 (0.04 to 1.97)		.04^g^		0.18
**GAD-7** ^h^ **(intervention: n=128; control: n=187)**									
	T1	6.45 (4.26)		6.49 (4.23)	—		—		—
	T3	5.09 (4.21)		5.42 (4.18)	0.39 (–0.46 to 1.07)		.43		0.08
	T4	4.49 (4.14)		5.20 (4.22)	0.40 (–0.09 to 1.48)		.08		0.17

^a^SD: subthreshold depression.

^b^T1: screening; T3: postevaluation; T4: 4-week follow-up.

^c^PSQI: Pittsburgh Sleep Quality Index.

^d^Not applicable.

^e^PAS: Panic and Agoraphobia Scale.

^f^PHQ-9: Patient Health Questionnaire-9.

^g^*P*<.05.

^h^GAD-7: Generalized Anxiety Disorder-7.

**Table 6:** Secondary outcome ANOVA results for the SI^a^ group.

Variables and weeks^b^			Intervention, mean (standard deviation)	Control group, mean (standard deviation)		SE (95% CI)		*P* value	Cohen's d
**CESD** ^c^ **(intervention: n=193; control: n=230)**									
	T1	15.74 (7.74)		15.45 (7.18)	—^d^		—		—
	T3	15.37 (7.99)		15.68 (8.46)	0.57 (–0.59 to 1.65)		.36		0.04
	T4	14.49 (8.20)		15.02 (8.48)	0.60 (–0.44 to 1.92)		.22		0.06
**PAS** ^e^ **(intervention: n=53; control: n=54)**									
	T1	3.91 (3.47)		3.43 (2.35)	—		—		—
	T3	4.74 (4.72)		4.80 (4.59)	0.86 (–1.37 to 2.03)		.70		0.01
	T4	4.26 (4.23)		3.83 (4.14)	0.78 (–1.75 to 1.34)		.79		0.10
**PHQ-9** ^f^ **(intervention: n=193; control: n=68)**									
	T1	3.98 (3.62)		5.01 (4.45)	—		—		—
	T3	4.53 (4.27)		5.10 (4.85)	0.63 (–0.67 to 1.82)		.37		0.13
	T4	4.13 (4.57)		4.19 (4.50)	0.65 (–1.21 to 1.35)		.91		0.01
**GAD-7** ^g^ **(intervention: n=193; control: n=230)**									
	T1	4.36 (4.13)		3.73 (3.46)	—		—		—
	T3	3.54 (3.94)		3.54 (3.88)	0.31 (–0.22 to 0.99)		.22		0.00
	T4	3.16 (3.68)		2.95 (3.68)	0.30 (–0.45 to 0.72)		.65		0.06

^a^SI: subthreshold insomnia.

^b^T1: screening; T3: postevaluation; T4: 4-week follow-up.

^c^CESD: Center for Epidemiologic Studies Depression Scale.

^d^Not applicable.

^e^PAS: Panic and Agoraphobia Scale.

^f^PHQ-9: Patient Health Questionnaire-9.

^g^GAD-7: Generalized Anxiety Disorder-7.

**Table 7:** Secondary outcome ANOVA results for the SP^a^ group.

Variables and weeks^b^			Intervention, mean (standard deviation)	Control group, mean (standard deviation)		SE (95% CI)		*P* value	Cohen's d
**PSQI** ^c^ **(intervention: n=99; control: n=98)**									
	T1	7.30 (2.76)		7.01 (2.79)	—^d^		—		—
	T3	7.27 (2.98)		6.91 (2.93)	0.28 (–0.68 to 0.42)		.64		0.12
	T4	6.80 (2.88)		6.73 (2.93)	0.28 (–0.39 to 0.72)		.56		0.02
**CESD** ^e^ **(intervention: n=99; control: n=98)**									
	T1	24.68 (10.23)		21.80 (10.26)	—		—		—
	T3	23.22 (9.53)		20.53 (9.21)	1.00 (–2.90 to 1.07)		.36		0.29
	T4	20.33 (8.98)		20.51 (9.48)	1.02 (–0.16 to 3.87)		.07		0.02
**PHQ-9** ^f^ **(intervention: n=99; control: n=98)**									
	T1	6.04 (4.96)		4.92 (4.70)	—		—		—
	T3	8.68 (5.83)		7.68 (5.71)	0.83 (–2.70 to 0.59)		.21		0.17
	T4	8.01 (5.47)		7.30 (5.31)	0.78 (–2.35 to 0.72)		.30		0.13
**GAD-7** ^g^ **(intervention: n=99; control: n=98)**									
	T1	8.55 (4.64)		7.44 (3.96)	—		—		—
	T3	7.26 (4.87)		6.28 (4.08)	0.52 (–1.32 to 0.73)		.57		0.22
	T4	5.95 (4.23)		5.76 (3.98)	0.48 (–0.52 to 1.37)		.38		0.55

^a^SP: subthreshold panic.

^b^T1: screening; T3: postevaluation; T4: 4-week follow-up.

^c^PSQI: Pittsburgh Sleep Quality Index.

^d^Not applicable.

^e^CESD: Center for Epidemiologic Studies Depression Scale.

^f^PHQ-9: Patient Health Questionnaire-9.

^g^GAD-7: Generalized Anxiety Disorder-7.

### Calculation of the MIC

Table 8 presents the MICs of the CESD, PSQI, and PAS. The mean MIC of the CESD for participants who undertook the Mentre program for SD was –5.23 (standard deviation 8.89). The mean MIC of the PSQI for participants who undertook the Mentre program for SI was –3.60 (standard deviation 6.34). The mean MIC of the PAS for participants who undertook the Mentre program for SP was –2.08 (standard deviation 2.44).

**Table 8:** MIC^a^ of each primary endpoint.

CESD^b^ in the SD^c^ group				PSQI^d^ in the SI^e^ group			PAS^f^ in the SP^g^ group	
MIC	Participants, n (%)	Mean (standard deviation)	Participants, n (%)		Mean (standard deviation)	Participants, n (%)		Mean (standard deviation)
Better	23 (16.0)	–9.78 (6.381)	18 (8.4)		–3.78 (2.184)	11 (11.8)		–7.91 (5.991)
Slightly better	48 (33.3)	–5.23 (8.890)	71 (33.2)		–2.08 (2.436)	45 (48.4)		–3.60 (6.340)
About the same	66 (45.8)	–3.61 (7.831)	113 (52.8)		–1.1 (1.911)	33 (35.5)		–1.79 (5.732)
Slightly worse	4 (2.8)	–1.00 (1.414)	11 (5.1)		0.91 (2.548)	4 (4.3)		–3.00 (4.761)
Worse	3 (2.1)	8.00 (14.731)	1 (0.5)		0.00	0		—^h^
Total	144 (100.0)	–4.82 (8.470)	214 (100.0)		–1.59 (2.338)	93 (100.0)		–3.44 (6.220)

^a^MIC: minimally important change.

^b^CESD: Center for Epidemiologic Studies Depression Scale.

^c^SD: subthreshold depression.

^d^PSQI: Pittsburgh Sleep Quality Index.

^e^SI: subthreshold insomnia.

^f^PAS: Panic and Agoraphobia Scale.

^g^SP: subthreshold panic.

^h^Not applicable.

### Adverse Events

None of the participants in either group experienced serious adverse events during the time of this study.

## Discussion

### Principal Findings

This study aimed to assess the effectiveness of the Mentre program, a 4-week ICBT, for SD, SI, and SP. We categorized participants into SD, SI, and SP groups based on their eligibility using previous research and conducted an RCT for each group. We verified differences in the score change from screening (T1) to 4-week follow-up (T4) as indicators of ICBT effectiveness. There was a significant difference between the SD and SI intervention and control groups but not in the SP groups. Additionally, significant differences in CESD score changes were observed from screening (T1) to postintervention (T3) in the SD intervention and control groups. The SI intervention and control groups also showed significant PSQI score changes from T1 to T3. Previous studies have yielded mixed results on ICBT for SD, indicating its effectiveness compared to usual treatment, with effectiveness maintained for more than 6 months [6,35]. Limited research on SI has also suggested the effectiveness of ICBT in improving insomnia symptoms [18]. Our findings of SD and SI are comparable to those of previous studies. However, there was no significant difference in PAS score changes in the SP intervention and control groups. One reason was the possibility that the eligibility of SP was not appropriate. Although no studies have yet properly defined and intervened on SP [36-40], a previous study [36] suggests that if agoraphobia is present, we should carefully attend to it even if it does not completely meet the panic disorder criteria. In another study [40], a participant was classified according to the severity based on the frequency of panic attacks. Our study participants were considered to have SP if they had no panic attacks and the duration of symptoms was less than 1 month but they scored more than 9 points on the PAS. Therefore, the Mentre program for SP did not work, because it focused on anticipatory anxiety or dealt with avoidance of a particular place.

At least in primary care, our Mentre app found that self-understanding depressive moods and thoughts and trying to change them into rational behavior are effective in SD and that recording and reviewing sleep are effective in SI. However, further research is needed for the definition of SP; in particular, it is necessary to verify whether ICBT programs, such as Mentre, are effective for SP accompanied by agoraphobia and panic attacks.

Regarding subthreshold criteria, it is not just SP that requires discussion. Previous studies [41,42] have emphasized the need to define the score indicating a healthy state for the definition of subthreshold symptoms. In our study, we considered the persistence of each subthreshold symptom for 2 weeks (T2), resulting in 458 participants no longer meeting the subthreshold criteria. Can we conclude that the 458 participants did not have subthreshold symptoms? It is worth considering whether the absence of symptoms for 2 weeks defines health. These patients were excluded from our study; however, we should consider the timing of the intervention for subthreshold symptoms, including medical histories, event factors, and the frequency of panic attacks or depressive episodes, in the definition of subthreshold symptoms in the future.

Given that the definition of the subthreshold state is ambiguous, it is difficult to estimate the appearance rate of subthreshold symptoms. In our study, we estimated 160 participants in each group as the target sample size, but in fact, there was considerable discrepancy. This may indicate that there are many more individuals with subthreshold symptoms than we predicted, possibly because there is substantial overlap across subthreshold states. In contrast, we also need to consider that the participants earned reward points by registering over the internet, which may relate to the high dropout rate, and the participants’ motivation to test the Mentre program should also be considered. Individuals with subthreshold symptoms may lack high motivation for treatment compared to diagnosed patients, because their symptoms are of a low level and do not impair everyday function. The Mentre program operates at the same level used for patients diagnosed with depression, insomnia, and panic disorder. Hence, daily participation may have been bothersome for the participants.

Future studies should be designed considering the concept of subthreshold symptoms and possible overlap across subthreshold conditions and having a structure that is manageable and appropriate for this particular patient population.

An essential consideration is the overlap across subthreshold symptoms. In this study, we verified all 3 subthreshold symptoms together, given the frequent comorbidity of depression and anxiety disorders, often with insomnia [43,44]. However, few previous studies have explored this overlap. A previous study [45] on universal prevention treatment for depression suggested that adolescents at low risk for depression benefit equally from preventive interventions as those at high risk; however, it did not specify a matching treatment for low risk [45]. In other words, patients with subthreshold symptoms may benefit from treatment, but the treatment is not specified, and a completely new approach may be necessary [46]. We hypothesize that if there is overlap across subthreshold symptoms, interventions targeting specific symptoms might also affect overlapping symptoms owing to their low severity. Unfortunately, our program only showed efficacy for the symptoms we pretargeted, but previous meta-analyses have shown that improvement in diagnosed insomnia is related to improved depressive symptoms [14]. Therefore, treatments focusing on identifying components of subthreshold symptoms and supplementing CBT techniques may be effective.

Additionally, our study provided evidence of the MIC for SD, SI, and SP, which is specific to the subthreshold condition. Despite being useful as an index for clinicians and patients, caution is necessary in generalizing to individuals, because the MIC provides a statistical measure of association and may not reflect clinical conditions [47]. Particularly, subthreshold symptoms were originally low in severity; hence, the MIC was also low. For the indicated MIC to be an important change, it is necessary to also examine the events and satisfaction levels that changed because of the MIC for patients who reported that they felt “slightly better.”

### Limitations

Several limitations of this study should be acknowledged. First, the participants were limited to internet research monitors; hence, it is possible that our sample may not have been representative of all individuals with subthreshold symptoms. Approximately 30% of the participants were not employed and had a low income, which also does not reflect the entire population. Second, the main analysis was a complete case analysis owing to concerns regarding the high dropout rate among patients with subthreshold symptoms. In this study, we needed to verify the effectiveness of the Mentre program for specialized subthreshold symptoms as a pilot study. In the future, more accurate efficacy verification research is needed by conducting RCTs on the general population and analyzing them using intent-to-treat methods.

### Conclusion

Our study suggests that the use of the 4-week Mentre program as a nonguided ICBT is associated with improvements in symptoms of SD and SI in primary care. However, for SP, there is a need to first define it, and next, it is necessary to re-examine the effectiveness of ICBT. Additionally, although the MIC of subthreshold symptoms may be useful in clinical situations, individual adaptation needs to be approached with caution.

## Appendix

Multimedia Appendix 1 CONSORT (Consolidated Standards of Reporting Trials) checklist.

## Abbreviations

CBT: cognitive behavioral therapy
CESD: Center for Epidemiologic Studies Depression Scale
DUI: duration of untreated illness
GAD-7: Generalized Anxiety Disorder-7
ICBT: internet-delivered cognitive behavioral therapy
MIC: minimally important difference
PAS: Panic and Agoraphobia Scale
PHQ-9: Patient Health Questionnaire 9
PRO: patient-reported outcome
PSQI: Pittsburgh Sleep Quality Index
RCT: randomized controlled trial
SD: subthreshold depression
SI: subthreshold insomnia
SP: subthreshold panic

## Data Availability

Raw data were generated at the research center of child mental developmental. Derived data supporting the findings of this study are available from the corresponding author (KT) upon request.
